# Improving Community Health Worker performance by using a personalised feedback dashboard for supervision: a randomised controlled trial

**DOI:** 10.7189/jogh.08.020418

**Published:** 2018-12

**Authors:** Caroline Whidden, Kassoum Kayentao, Jenny X Liu, Scott Lee, Youssouf Keita, Djoumé Diakité, Alexander Keita, Samba Diarra, Jacqueline Edwards, Amanda Yembrick, Isaac Holeman, Salif Samaké, Boureima Plea, Mama Coumaré, Ari D Johnson

**Affiliations:** 1 Muso, Bamako, Mali, and San Francisco, California, USA; 2 Malaria Research & Training Center, University of Sciences Techniques and Technologies of Bamako, Mali; 3 University of California San Francisco, Department of Social and Behavioral Sciences, San Francisco, California, USA; 4 Harvard Medical School, Boston, Massachusetts, USA; 5 Medic Mobile, San Francisco, California, USA; 6 Malian Ministry of Health and Public Hygiene, Bamako, Mali; 7 University of California San Francisco, ZSFG Division of Hospital Medicine, San Francisco, California, USA

## Abstract

**Background:**

Countries across sub-Saharan Africa are scaling up Community Health Worker (CHW) programmes, yet there remains little high-quality research assessing strategies for CHW supervision and performance improvement. This randomised controlled trial aimed to determine the effect of a personalised performance dashboard used as a supervision tool on the quantity, speed, and quality of CHW care.

**Methods:**

We conducted a randomised controlled trial in a large health catchment area in peri-urban Mali. One hundred forty-eight CHWs conducting proactive case-finding home visits were randomly allocated to receive individual monthly supervision with or without the CHW Performance Dashboard from January to June 2016. Randomisation was stratified by CHW supervisor, level of CHW experience, and CHW baseline performance for monthly quantity of care (number of household visits). With regression analysis, we used a difference-in-difference model to estimate the effect of the intervention on monthly quantity, timeliness (percentage of children under five treated within 24 hours of symptom onset), and quality (percentage of children under five treated without protocol error) of care over a six-month post-intervention period relative to a three-month pre-intervention period.

**Results:**

Use of the Dashboard during monthly supervision significantly increased the mean number of home visits by 39.94 visits per month (95% CI = 3.56-76.3; *P* = 0.031). Estimated effects on secondary outcomes of timeliness and quality were positive but not statistically significant. Across both study arms, CHW quantity, timeliness, and quality of care significantly improved over the study period, during which time all CHWs received dedicated monthly supervision, although effects plateaued over time.

**Conclusions:**

Our findings suggest that dedicated monthly supervision and personalised feedback using performance dashboards can increase CHW productivity. Further operational research is needed to understand how to sustain the performance improvements over time.

**Trial registration:**

ClinicalTrials.gov (NCT03684551).

Community Health Workers (CHWs) have been shown to improve access to care and reduce maternal, newborn, and child morbidity and mortality [1-3]. Investment in CHW-led health systems is experiencing a resurgence in the era of Sustainable Development Goals, with countries across sub-Saharan Africa scaling CHW programmes as an evidence-based strategy for achieving universal health coverage [4].

It has widely and long been acknowledged in the literature that supervision is a necessary pillar of successful CHW programmes [5-7]. Yet, it is one of the most overlooked features in the design and implementation of CHW programmes, with considerable consequences. Qualitative research with CHWs in a number of different contexts has found that supervision is infrequent and irregular, unsupportive, and ultimately ineffective. CHWs cite a lack of frequent and regular supportive supervision as negatively affecting their job satisfaction and motivation, [8-11] retention, [7,12] and performance [13].

Few strategies for CHW supervision, or their specific components, have been tested [14]. Specifically, there remain unanswered questions with respect to who provides supervision, where, at what frequency, with what content, and with what tools. Among 80 studies where a supervision structure was mentioned in a systematic review of design features that influence CHW performance, most lacked information on its precise design and implementation [15]. One study in Madagascar showed that less frequent supervision resulted in lower CHW performance, while another in Kenya found no effect of frequency on CHW guideline adherence [15].

This lack of attention to and investment in supervision could compromise national-scale CHW programmes that are being designed and implemented in countries across sub-Saharan Africa. Recent independent evaluations in Burkina Faso, Ethiopia, and Malawi found inadequate CHW supervision and low CHW performance in national-scale integrated Community Case Management (iCCM) of common childhood illnesses [16-18]. Ultimately, these studies of national iCCM scale up found no significant impact on care-seeking or under-five child mortality. There is an urgent need for practitioners and policymakers to know how to design and implement CHW supervision that leads to higher quality CHW performance and ultimately to the desired effects of CHW programmes on population health.

In this study, we examined the effectiveness of a mobile health technology (mHealth) tool for personalised performance feedback during CHW supervision on CHW performance. Evidence on the effectiveness of mHealth tools for improving CHW performance is limited; two reviews found randomised and observational evidence that mHealth tools improve the quality of CHW services, but conclude that a stronger evidence base is needed to inform policy and practice [19,20]. Specifically, a recent systematic review of interventions for improving CHW performance found moderate quality evidence that when supervising CHWs, escalating SMS reminders for tasks that are overdue improved CHW performance [21].

This randomised controlled trial used a dedicated cadre of CHW supervisors, recruited and trained for the exclusive purpose of supervising CHWs in the peri-urban area of Yirimadio in Bamako, Mali. CHWs were randomised to receive individual monthly supervision from their dedicated supervisor with or without the aid of the CHW Performance Dashboard – an mHealth personalised performance feedback tool, which graphically displays a CHW’s monthly performance in terms of quantity, timeliness, and quality of care provided alongside those of the highest performing CHW. Using longitudinal data, our analysis sought to determine the effect of the Dashboard on CHW performance improvement in terms of the quantity, timeliness, and quality of care provided.

## METHODS

### Study design

**Figure 1:**
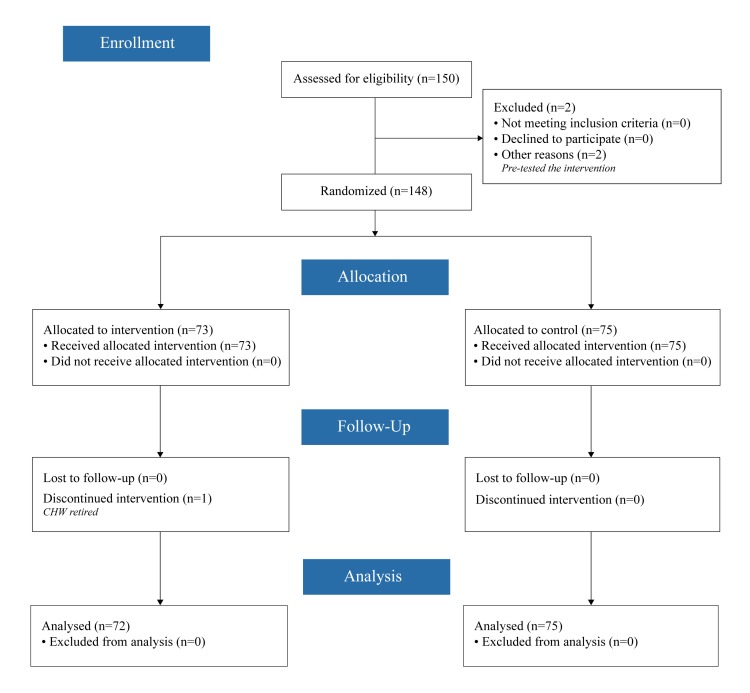
CONSORT diagram.

This randomised controlled trial (see **Figure 1** for CONSORT diagram) was designed to test the effect of an individualised performance dashboard visual feedback tool used during supervisory sessions on CHW performance improvement. The ICMJE defines a clinical trial as “any research study that prospectively assigns human participants or groups of humans to one or more health-related interventions to evaluate the effects on health outcomes.” At the study outset, the investigators did not consider CHW performance metrics to be health outcomes, as these are not at the patient level nor are they biomedical, pharmacokinetic measures, or adverse events. After further consideration, the study investigators decided to adopt the broader definition of clinical trial and registered the trial retrospectively at ClinicalTrials.gov (NCT03684551).

### Study setting and participants

This study was conducted in Yirimadio, a peri-urban area of Bamako, Mali with an estimated population of 163 500 in 2016 [22]. Yirimadio is the site of a CHW-led health systems strengthening intervention, called proactive community case management (ProCCM), jointly launched in 2008 by a nongovernmental organisation and the Malian Ministry of Health and Public Hygiene (full description of the intervention is published elsewhere) [23]. During the study period of October 2015 to June 2016, 150 CHWs were employed in non-overlapping intervention zones covering all of Yirimadio to provide health services to these communities. These CHWs were supervised by eight dedicated CHW supervisors, recruited and trained for the exclusive purpose of supervising the 15 to 20 CHWs under each of their supervision. All eight supervisors and 150 CHWs participated in the current study: 148 CHWs were study participants providing written informed consent, while two CHWs pretested the Dashboard.

### Description of the intervention

**Figure 2:**
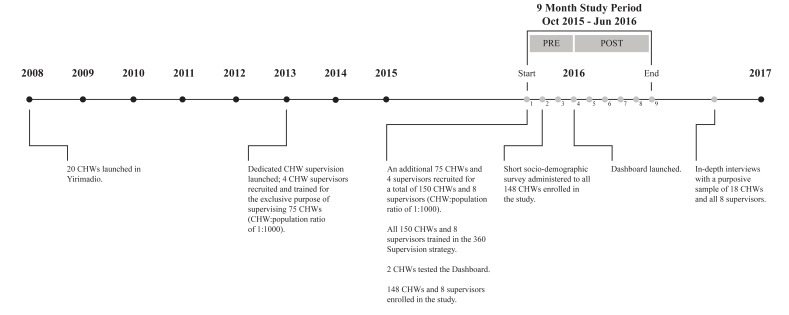
Timeline of the intervention and related research activities.

A timeline of the intervention and related research activities are summarized in **Figure 2****.**

#### The CHW-led health system

During the study period, all CHWs, regardless of treatment arm, performed proactive case detection, the process of conducting at least two hours per day of door-to-door home visits to proactively identify—through health history inquiry and/or disease diagnostics—patients who need care. For all patients identified, CHWs provided doorstep counselling, evaluation, diagnostics, treatment, referral to appropriate health facilities, and follow-up (full description provided in **Online Supplementary Document(Online Supplementary Document):** *Description of the CHW-led health system strengthening intervention*). CHWs provided care in the community without user fees, and were able to refer patients to the reinforced government primary health centres for care without user fees as well. CHWs were residents of the communities they served, and they were required to be available at home or by phone for consultation at any time. All CHWs were compensated with a monthly salary of 40 000 FCFA (approx. US$ 70) plus 1000 FCFA (approx. US$ 2) in mobile phone communication credit for this part-time work.

#### The CHW supervision model

Eight CHW supervisors were recruited (four in 2013, four in October 2015) and all trained (in October 2015) for the exclusive purpose of each supervising 15 to 20 CHWs using a monthly dedicated supervisory strategy called 360 Supervision. CHWs in both study arms received monthly individual supervisory sessions (lasting approximately three hours) and weekly group supervisory sessions (lasting approximately two hours) from their dedicated CHW supervisor. At weekly group sessions, which brought together CHWs in both intervention and control arms, the supervisor led discussion of the common challenges and potential solutions faced by CHWs. During the final group session of each month, the supervisor and CHWs agreed on a schedule of individual supervision sessions for the coming month. Each CHW knew in advance the date and time, but not the location within his/her intervention zone at which the next individual supervision would take place.

**Figure 3:**
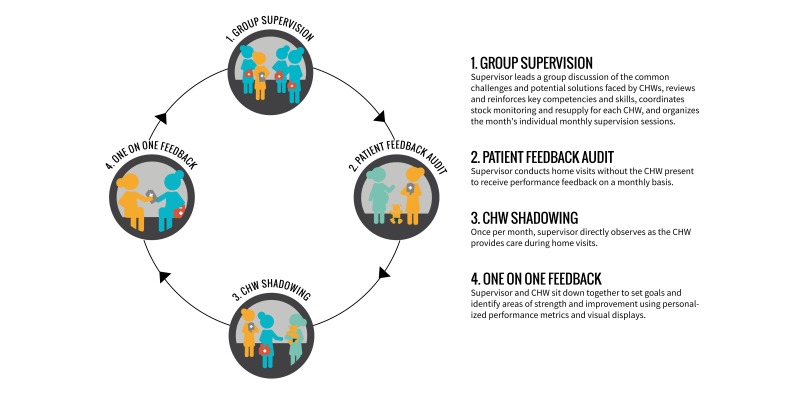
360 Supervision strategy.

An individual monthly session of 360 Supervision included: (i) solicitation of patient perspectives of CHW care; (ii) direct observation of CHW doorstep care; and (iii) a one-on-one feedback discussion (**Figure 3**), with or without the CHW Performance Dashboard depending on treatment arm. On the scheduled day of a CHW’s individual supervision, the supervisor chose an area (different each month) within the CHW’s zone to conduct home visits in the absence of the CHW for the purpose of soliciting patient perspectives of CHW care and verifying CHW reporting. Supervisors interviewed the female head of household or her representative using a paper-based data collection form to record: knowledge of the CHW (ie, contact information); frequency of CHW visitation; type and quality of CHW services received; suggestions for improvement. After soliciting patient perspectives for at least one hour, the supervisor and CHW met for at least one hour of direct observation. At each home visit conducted by the CHW, the supervisor used another paper-based form to record his/her observations on: CHW behaviour/demeanour; questions asked and information provided; adherence to protocol; duration of the visit; maintenance of supplies; completion of patient care forms. Finally, the supervisor and CHW then sat together privately for approximately 45 minutes for a one-on-one feedback discussion of the CHW’s strengths and areas for improvement, guided by another paper-based form and informed by the information recorded during the first two phases of individual supervision.

#### The CHW Performance Dashboard

**Figure 4:**
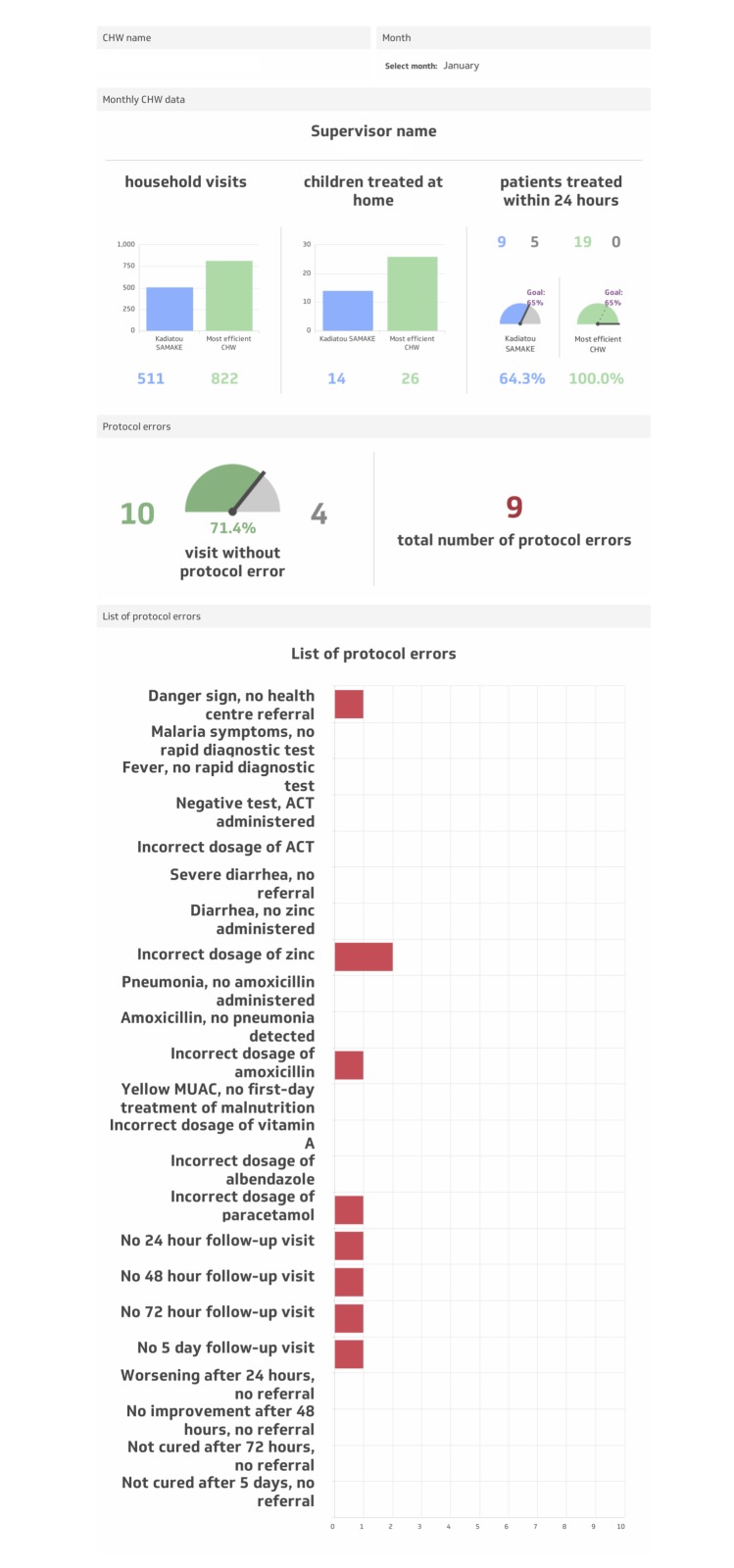
CHW Performance Dashboard, English translation.

For CHWs randomised to the intervention arm, a visual feedback tool, the CHW Performance Dashboard, was employed during the individual supervisory feedback session (phase three of monthly supervision), starting in January 2016. The CHW Performance Dashboard (**Figure 4**) was a graphic display of a CHW’s performance along three indicators defined as follows:

- “Quantity” of care: the number of homes visited during the month;
- “Timeliness” of care: the percentage of sick children under five treated within 24 hours of symptom onset during the month (during proactive case-finding home visits, CHWs recorded the date and time of day of the visit—morning, noon, evening, and night. Based on the parent/guardian’s recall of the time of day of symptom onset (ie, today, over the course of the night, yesterday morning, yesterday evening, day before yesterday morning, day before yesterday evening, three days ago, or more than three days ago), a dichotomous variable was created to indicate if the visit took place within 24 hours of symptom onset);
- “Quality” of care: the percentage of sick children under five treated without protocol error among 23 potential errors during the month.

The Dashboard displayed an individual CHW’s quantity, timeliness, and quality of care indicators from the previous month, using absolute numbers, percentages, and visual graphics, alongside those of the highest performing CHW. During the individual supervisory feedback session, this personalised and relative (to the highest performer) quantitative performance feedback helped orient the discussion of strengths and weaknesses, and allowed the CHW to see quantitatively and visually how his/her performance fared the previous month. The feedback provided to CHWs in the intervention arm, therefore, was both quantitative, informed by the Dashboard, and qualitative, informed by the patient perspectives (phase one) and direct observation (phase two).

The supervisory feedback session for CHWs in the control arm was not facilitated by the Dashboard or any personalised quantitative feedback on quantity, speed, or quality of care. CHW supervisors were instructed to continue providing CHWs in the control arm with feedback informed by patient perspectives (phase one) and direct observation (phase two).

The primary outcome of quantity of services provided, and the secondary outcomes of timeliness and quality of services provided, were followed longitudinally from January (when the Dashboard was introduced) to June 2016. Three metrics were chosen to provide a holistic measure of CHW performance.

### Randomisation

Randomisation of 148 individual CHWs (73 intervention; 75 control) was stratified by three variables: (i) CHW supervisor, to ensure that each had equal numbers of CHWs from both arms; (ii) level of CHW experience as determined by number of years as a CHW (a dichotomous variable for being recruited in 2008 or 2013, vs recruited in 2015); and (iii) CHW pre-intervention quantity of care performance over the previous 12 months (for 2008- and 2013-recruited CHWs) or over the previous two months (for 2015 recruited CHWs) per the estimated coefficients from a cubic trend (ie, *y_i_ = a + bx_i_+cx_i_^2^+ dx_i_^3^+e_i_ for each CHW i*). CHWs were not blinded to their randomisation assignment.

### Source data

A short survey collecting basic socio-demographic information was administered in the local language, Bamanankan, to all participating CHWs in November 2015. Over the course of the study, CHWs recorded all proactive case-finding home visits using a specific paper form, and all community case management of children under five using a different paper form. Data forms were collected weekly by supervisors at the group supervision meetings. Supervisors checked for data completion; CHWs were asked to revisit households to complete missing data. The validity of source data was checked through a monthly internal telephone audit, a process by which supervisors randomly selected two households from among the proactive case-finding records and one patient care record for each CHW to phone and confirm visit information with the household representative, patient, or patient’s guardian. No CHW was found to be falsifying data over the course of the study.

Each week, supervisors transferred data forms to data clerks, who were blinded to randomisation, responsible for entering the data into two separate Microsoft Excel databases (Microsoft Inc, Seattle, WA, USA). CHWs (in both study arms) were again asked to revisit households to complete any missing data identified by the data clerks. At the end of each month, the data manager compiled the Excel databases from different data clerks, generated queries to correct for data entry errors, and uploaded the data files to the cloud-based application (Klipfolio), upon which the data analysed for all CHWs in this study is based and from which monthly CHW Performance Dashboards were generated for CHWs in the intervention arm. It should be noted that, while the Dashboard was used in the intervention arm for each month of the post-intervention period, there were operational delays, particularly towards the end of the study period, in getting the Dashboards ready, printed, and distributed to supervisors. Thus, in latter months of the study, supervisors had less time during the month to complete their supervisory activities.

### Data preparation

Data were prepared and analysed in Stata Version 12 (State Inc, College Station, TX, USA). Monthly CHW patient data, CHW socio-demographic data, and CHW randomisation assignment were merged together. One (intervention) CHW was removed from all analyses because she left her post in February 2016. For CHWs who worked fewer than 15 days in one month (eg, due to pregnancy, disease, family death), their quantity data was replaced with missing for that month only, as the absolute number of home visits that a CHW can accomplish in less than half of a month is not an appropriate reflection of a CHW’s performance.

### Descriptive analyses

To assess the validity of randomisation, we first tested for differences in baseline CHW socio-demographic characteristics between the intervention and control arms. We descriptively examined the distribution of our outcome variables and the extent of the variation within and between CHWs. The mean monthly values for each outcome by study arm were graphed to visualise the average performance trends.

### Regression analyses

We conducted two main regression analyses. To test for an overall improvement on outcomes across both study arms over the entire time period (October 2015 - June 2016), we ran the following model (**Equation 1**):

*y_im_ = a+β_1_T_i_+β_2_Z_m_+β_3_Z_m_^2^+e_im_*,

where *y_im_* is an outcome of interest (ie, quantity, timeliness, quality) for CHW *i* at time *m*; *T_i_* is an indicator for treatment assignment to the Dashboard intervention arm; both *Z_m_*, a linear monthly time trend, and *Z_m_^2^*, a quadratic monthly time trend, are included to allow for flexibility in the functional form; and *e_im_* is the idiosyncratic error term. The coefficients of interest are *β_1_* and *β_2_* which provide an estimate of the time trend trajectory of the outcome over the entire study period.

In order to test for the main effect of the Dashboard intervention, we estimated the following difference-in-difference model (**Equation 2**):

y_im_ = a+β_1_T_i_+β_2_P_m_+β_3_T_i_ × P_m_+μ_m_+e_im_

where *y_im_* is an outcome of interest (ie, quantity, timeliness, quality) for CHW *i* at month *m*; *T_i_* is an indicator for treatment assignment to the Dashboard intervention arm; *P_m_* is a dummy variable for the post-intervention period (January – June 2016), *μ_m_* represents a vector of month fixed effects; and *e_im_* is the idiosyncratic error term. The coefficient of interest is *β_3_* on the interaction term representing the mean difference between treatment and control arms in the post-intervention period as compared to the pre-intervention period (October – December 2015). All regressions included random effects by CHW; standard errors were clustered by CHW. In additional sensitivity analyses, we examined the robustness of our result to (a) alternative methods for controlling for time (ie, continuous trends), (b) account for unobservable heterogeneity by including fixed effects for individual CHWs, and (c) examine more efficient estimation approaches via ANCOVA (Hausman tests showed that the inclusion of CHW fixed effects did not significantly improve the consistency of point estimates over the efficiency gained with random effects).

### Qualitative interviews

We conducted individual in-depth interviews in October 2016 with a purposive sample of 18 CHWs (the least, average, and highest performing CHW from each study arm for each indicator) and all eight CHW supervisors to elucidate the possible mechanisms of action of the Dashboard on CHW performance. Interviews were conducted in the local language, Bamanankan, and included structured, open-ended questions on 360 Supervision for both intervention and control CHWs, and structured, open-ended questions on the Dashboard for supervisors and intervention CHWs. Each interview was recorded, transcribed, and translated into French by an independent consultant, who conducted qualitative analysis with Atlas.ti (Scientific Software Development GmbH, Berlin, Germany) to identify key themes.

## RESULTS

### Sample characteristics and randomisation balance

**Table 1** displays the characteristics of CHWs involved in this study. Mean CHW age was 33 years. One third (33%) had attended at least secondary level education. CHWs were well established within their community, having lived in Yirimadio for a mean of 14 years. Tests for differences in mean characteristics between the control and intervention arms indicated that CHWs were not significantly different along any of the observed socio-demographic or performance indicators at baseline.

**Table 1:** CHW socio-demographic characteristics at baseline in the intervention and control arms

Sociodemographic Characteristic		All frequency (%) (n = 147)		Intervention frequency (%) (n = 72)	Control frequency (%) (n = 75)		*P*
**Age in years:**							
Mean (SD)	33.7 (9.9)		34.1 (10.0)			33.3 (9.8)	0.648
**Highest level of school attended:**							
Primary	86 (59)		42 (58)			44 (59)	0.755
Secondary	48 (33)		25 (35)			23 (31)	
Tertiary	13 (9)		5 (7)			8 (11)	
**Marital status:**							
Single	19 (13)		9 (13)			10 (13)	0.915
Married	119 (81)		58 (81)			61 (81)	
Divorced/widowed	9 (6)		5 (7)			4 (5)	
**Household size:**							
Mean (SD)	8.1 (4.2)		8.1 (3.5)			8.0 (4.8)	0.856
**Years of experience as a CHW:**							
2+ years	75 (51)		34 (47)			41 (55)	0.367
None	72 (49)		38 (53)			34 (45)	
**Length of time living in intervention zone (years):**							
Mean (SD)	14.0 (9.2)		14.0 (9.1)			14.0 (9.3)	0.986
1-10	57 (39)		28 (39)			29 (39)	0.932
11-20	59 (40)		38 (39)			31 (41)	
>20	31 (21)		16 (22)			15 (20)	

CHW – Community Health Worker, SD – standard deviation

### Overall trends in performance

**Figure 5:**
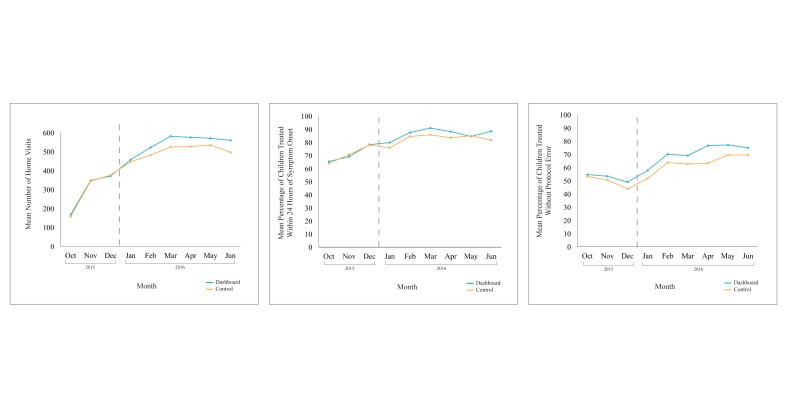
Mean quantity (left), timeliness (middle), and quality (right) performance trends during pre- (left of dotted line) and post-intervention (right of dotted line) periods for CHWs in the control and intervention arms.

CHW performance on all three outcomes improved over the study period. For all CHWs, the average quantity, timeliness, and quality of care was greater in the post-intervention period compared to the pre-intervention period (**Table 2**): On average, CHWs conducted more proactive case-finding home visits per month (522 vs 294), treated a higher percentage of sick children under five within 24 hours of symptom onset per month (85% vs 71%), and treated a higher percentage of children under five without protocol error per month (67% vs 50%) in the post-intervention period compared to the pre-intervention period. In the post-intervention period, CHWs in the intervention arm also performed better on average than CHWs in the control arm, in terms of quantity (544 vs 501), timeliness (87% vs 83%), and quality (71% vs 63%) of care. These monthly trends on performance outcomes are displayed in **Figure 5**, showing overall improvements over time, but that performance plateaued in the post-intervention period.

**Table 2:** Descriptive statistics of CHW performance outcome variables during pre-intervention (3 months) and post-intervention (6 months) periods, for all CHWs and by treatment arm

Performance indicator	Mean (SD)		
**Pre-intervention period, Oct-Dec 2015**	**Post-intervention period, Jan-Jun 2016**	
**Quantity:**			
Number of home visits per month			
Control (n = 75)	291.8 (146.4)	501.4 (163.5)	
Treatment (n = 72)	294.6 (138.8)	544.1 (188.4)	
Overall (n = 147)	293.5 (142.3)	522.4 (177.3)	
**Timelines:**			
Percentage of children under five treated within 24 h of symptom onset			
Control (n = 75)	71.1 (25.6)	82.7 (25.0)	
Treatment (n = 72)	71.0 (29.5)	86.6 (22.5)	
Overall (n = 147)	70.7 (27.9)	84.6 (23.8)	
**Quality:**			
Percentage of children under five treated without protocol error			
Control (n = 75)	49.1 (25.3)		63.4 (29.1)
Treatment (n = 72)	52.3 (25.1)		71.0 (25.4)
Overall (n = 147)	50.4 (25.4)		67.1 (27.6)

CHW – Community Health Worker, SD – standard deviation

There was a significant upward, but plateauing, trend in quantity, timeliness, and quality performance for CHWs in both arms over the entire study period (**Table 3**; *P* < 0.001). For all three performance indicators, alternative specifications including higher order terms for the monthly time trend were conducted and yielded the same overall results.

**Table 3:** CHW performance trends over the nine-month study period, October 2015 to June 2016

	Quantity			Timelines			Quality		
	**Coef†**	**95% CI**	***P***	**Coef†**	**95% CI**	***P***	**Coef†**	**95% CI**	***P*-value**
Dashboard treatment	29.37	-3.40, 62.14	0.079	2.74	-2.45, 7.92	0.301	6.19*	1.59, 10.80	0.008
Month	138.20**	123.64, 152.77	<0.001	8.78**	6.32, 11.23	<0.001	2.33	-0.11, 4.78	0.062
Month-squared	-9.63**	-11.20, -8.05	<0.001	-0.62**	-0.84, -0.41	<0.001	0.09	-0.14, 0.32	0.435
Constant	43.93*	13.87, 73.99	0.004	54.70**	47.84, 61.57	<0.001	43.98**	38.17, 49.79	<0.001
F-test χ2‡		807.70**	0.000		71.90**	<0.001		128.93**	<0.001
R-sq.		0.3920			0.0834			0.1031	
CHWs		147			147			147	
Observations		1297			1277			1297	

Coef – coefficient, CI – confidence interval, CHW – community health worker

*P < 0.05, **P < 0.001.

†Estimates for a random effects panel regression using a quadratic time trend and a treatment dummy that takes value 0 for the control arm and value 1 for the intervention arm. All estimates are adjusted for clustering at the CHW level and heteroskedasticity using the *vce(cluster clustvar)* command in Stata.

‡Test of the joint significance of estimated coefficients on the linear and quadratic month terms.

### Treatment effects on performance

The estimated mean effect of the Dashboard intervention was a significant increase in the number of home visits per month by 39.94 (95% CI = 3.56-76.3; *P* < 0.05; **Table 4**). Although estimated effects on timeliness and quality were also positive, these estimated effects were not statistically significant. Sensitivity analyses showed that these point estimates were consistent across specifications that alternatively controlled for continuous time trends, included CHW fixed effects, and used an ANCOVA model (Table S1 in **Online Supplementary Document(Online Supplementary Document)**).

**Table 4:** Estimated mean effects of the dashboard intervention on CHW performance outcomes

	Quantity			Timelines			Quality		
	**Coef†**	**95% CI**	***P***	**Coef†**	**95% CI**	***P***	**Coef†**	**95% CI**	***P***
Treatment	3.00	-22.9, 28.9	0.821	-0.11	-6.66, 6.44	0.974	3.06	-2.15, 8.27	0.249
Post	341.68**	301.4, 381.9	<0.001	17.97**	11.2, 24.8	<0.001	15.33**	8.1 - 22.6	<0.001
Treatment × Post‡	39.94*	3.56, 76.3	0.031	4.21	-2.39, 10.8	0.211	4.56	-1.58, 10.7	0.145
CHWs		147			147			147	
Observations		1297			1277			1277	

Coef – coefficient, CI – confidence interval

*P < 0.05, **P < 0.001.

†All regressions include fixed effects by month. Standard errors are adjusted for heteroskedasticity and clustering at the CHW level.

‡The × in ‘Treatment × Post’ indicates an interaction between the variables ‘Treatment’ and ‘Post’.

### In-depth interviews

In qualitative interviews, all supervisors expressed their appreciation of the Dashboard as a tool which allowed them to be more effective in their supervision, citing its ability to facilitate one-on-one feedback with CHWs, track changes in individual CHW performance, and identify common difficulties experienced by CHWs in general. Both intervention and control CHWs cited the Dashboard (including specifically seeing the metrics of the highest performer) or the very idea of the Dashboard, respectively, as a source of motivation. Intervention CHWs indicated that the Dashboard was useful in identifying specific problems, both individual and general (eg, stock outs). Both intervention and control CHWs cited individual monthly supervision as a source of motivation and beneficial to their work, because their dedicated supervisor corrected errors made during direct observation and on patient forms, ensured their stocks and supplies, engaged reticent community members, and was available to answer questions.

## DISCUSSION

Despite the importance of supervision for effective CHW programmes, little research is available to inform policymakers and programme administrators about the functions and processes of supervision needed to optimise CHW performance. Our study shows that the CHW Performance Dashboard tool, a personalised visual display of absolute and relative performance feedback used during monthly supervision sessions, significantly increased the number of home visits conducted by CHWs without compromising timeliness or quality of care. This indicates that CHWs who received 360 Supervision with the Dashboard became more productive than CHWs supervised without the Dashboard.

This study was designed to capture the effects that the Dashboard might have had in influencing how supervisors and CHWs randomised to the intervention related during one-on-one supervision. However, there may have been positive spill-over effects due to the sharing of the same supervisors between intervention and control CHWs and the abundance of information that supervisors had about individual CHW performance. The decision to stratify randomisation by supervisor was intentional, as a means of controlling for confounding by supervisor, given the relatively small number of supervisors in our cohort. In retrospect, supervisors could have organised group supervision separately by study arm in an effort to minimise contamination. Supervisors may have taken what they learned through the Dashboard and translated that to the benefit of all CHWs (ie, during one-on-one supervision with control CHWs and/or during weekly group supervision which included CHWs from both arms together). Indeed, in ancillary qualitative interviews, supervisors reported that the Dashboards helped to improve the efficacy of their supervision in general, for example, by highlighting the most commonly detected protocol errors. Both intervention and control CHWs reported feeling “motivated” by the very idea of the Dashboard to improve performance. Finally, the Dashboard may have assisted in the detection of systemic issues that affect all CHWs. In particular, certain protocol errors (eg, detection of diarrhoea without the administration of zinc) resulted from nationwide stock outs rather than poor individual performance. Hence, the magnitude of our estimates of the Dashboard effects may be underestimated.

Despite our efforts to clarify the intervention’s mechanism of action through in-depth interviews, we cannot definitively distinguish among these explanations with the available data. However, qualitative evidence suggests that all of these alternative mechanisms of action were at play over the course of the study; this was unanticipated because control CHWs never received personalised performance dashboards. This finding is consistent with a theme observed elsewhere: that digital health interventions can be remarkably complex [24,25]. Undertaking extensive contextual, human-centred design work (eg, iteratively testing improvements to the tool) before proceeding to a controlled trial is one strategy for elucidating the myriad contextual factors and multiple simultaneous mechanisms of action [24,25].

Performance improvements were observed for all CHWs since the start of the study period (ie, before the Dashboard intervention was introduced), during which time all CHWs were receiving 360 Supervision. This study was not designed to test the overall effect of the 360 Supervision model, which precludes us from fully attributing performance improvements to this strategy. Nevertheless, it suggests that supervision design may influence CHW performance regardless of added benefit of the Dashboard tool.

Different design features of 360 Supervision may have improved performance for all CHWs. First, 360 Supervision was dedicated; it was ensured by a CHW supervisor recruited and trained specifically and exclusively for the job. The dedicated cadre of CHW supervisors in our study is a notable departure from many other contexts, where the ability of facility-based health professionals to provide satisfactory supervision to CHWs is circumscribed by challenges such as added work burden, distance and transport, and lack of training in supportive supervision [16,26-29]. By contrast, dedicated supervisors were available full-time to provide community-based supervision, thereby understanding the contextual realities of a CHW’s work environment. Second, 360 Supervision was frequent and regular; the dedicated supervisor-to-CHW ratio was such that individual supervision for every CHW could take place once per month. The monthly rate in our study provided recurring opportunities for CHWs and supervisors to address issues in service delivery. Additionally, frequent and regular contact may have facilitated the relationship between CHW and supervisor; both actors reported respectful, reliable, supportive relationships in qualitative interviews. Finally, 360 Supervision was holistic; it involved patient perspectives, direct observation of clinical skills, and one-on-one coaching on strengths and weaknesses. In qualitative interviews, CHWs from both arms cited the workflow as useful in identifying and addressing, in real-time, challenges faced and errors made in delivering health services.

Dedicated, monthly 360 Supervision is one part of the CHW-led health system intervention in this catchment area. Proactive community case management (ProCCM) was introduced in Yirimadio in 2008, with dedicated monthly supervision for CHWs added in 2013. By 2015, the under-five mortality rate in Yirimadio had dropped to 7 per 1000 live births, from a baseline rate of 155 per 1000 in 2008 (HR = 0.039, *P* < 0.0001) [23]. During this period, early access to effective treatment for childhood febrile illness more than doubled (OR = 3.198, *P* < 0.0001). This seven-year repeated cross-sectional study was observational, and therefore, no causal inference could be made attributing the changes in child mortality and access to care to the complex intervention or its specific components: (1) proactive case detection by CHWs, (2) CHW doorstep care, (3) dedicated monthly supervision, (4) removal of user fees, (5) improvements at the primary care centre in infrastructure and capacity. The performance improvements for all Yirimadio CHWs in the current study are equally observational, and the observations reported here were made after the declines in child mortality. Nevertheless, taken together in the context of existing evidence, the results of these studies suggest that dedicated monthly CHW supervision may improve CHW performance, lead to faster access to quality care, and ultimately reduce mortality.

It is important to note that the performance improvements observed for all CHWs in this study plateaued over time. A tapering effect of performance improvements was also recently found in India: CHWs using self-tracking analytics conducted 21.5% more patient visits per month than their control peers, but mean monthly visits declined in both arms over time – an effect that authors attribute to a scaling back of traditional supervision [30]. There are several possible explanations for the plateauing trends in our study. First, the operational delays in preparing the Dashboard in the latter months left supervisors with less time to perform their duties and may have reduced the quality of supervision. Second, supervisors could have lost motivation over time, which might have reduced the effectiveness of their supervision. Third, there may be a limit to the quantity, timeliness, and quality of care that is operationally feasible per CHW.

Our results highlight several areas that should be further studied in future research. These include: employing a mixed-methods, human-centred approach to iteratively test improvements to the Dashboard or related workflow to maximise impact; incorporating regular monitoring activities in order to understand process outcomes; collecting additional longitudinal data to understand the longer-run permanency of the intervention effects; testing the tool in the context of more time- and information-constrained (ie, facility-based) supervision models; and finally, conducting operational research to understand precisely how to design and implement CHW supervision more broadly – supervision by whom, with what frequency, and with what tools – in order to optimise CHW performance.

## CONCLUSIONS

Our findings suggest that using personalised performance feedback in dedicated monthly supervision can positively and substantively contribute to improved CHW performance without compromising the quality and timeliness of care. More operational research is needed to understand how to optimise CHW supervision and personalised performance dashboards, and how to sustain the performance improvements over time.
