# Supplementary material for: Improving Community Health Worker performance by using a personalised feedback dashboard for supervision: a randomised controlled trial
Source: J Glob Health. 2018 Oct 2;8(2):020418. doi: 10.7189/jogh.08.020418 (PMC6162089; doi:10.7189/jogh.08.020418)
Supplement: Online Supplementary Document [file jogh-08-020418-s001.pdf]

Online Supplementary Document

Whidden et al. Improving Community Health Worker performance by using a personalised feedback dashboard for supervision: a randomised controlled trial

J Glob Health 2018;8:020418

Table S1: Sensitivity analyses of the estimated mean effects of the dashboard intervention

	**Quantity**			**Timeliness**			**Quality**		
	Coef[1]	95% CI	p-value	Coef[1]	95% CI	p-value	Coef[1]	95% CI	p-value
**Panel A: Controlling for continuous time trends[2]**									
Treatment*Post	39.88*	3.57, 76.2	0.031	4.21	-2.37, 10.8	0.210	4.55	-1.60, 10.7	0.147
**Panel B: Inclusion of CHW fixed effects[3]**									
Treatment*Post	40.02*	3.34, 76.7	0.033	4.31	-2.35, 11.0	0.203	4.61	-1.60, 10.8	0.145
**Panel C: ANCOVA model[4]**									
Treatment*Post	40.52*	4.20, 76.85	0.029	4.52	-0.90, -9.94	0.102	6.19*	0.79, 11.6	0.035
Number of CHWs[2]		147			147			147	
Observations[3]		1297			1277			1277	

[1] For all specifications, standard errors are adjusted for corrected for heteroskedasticity and clustered at the CHW level.
[2] Includes controls for treatment arm, post-intervention period, and fixed effects by month.
[3] Includes fixed effects by month.
[4] Includes controls for the mean baseline (i.e., over the three pre-intervention months) value of the outcome variable and fixed effects by month.
[2] Quantity has fewer observations than timeliness or quality because CHWs who worked less than 15 days in one calendar month due to permitted leave (pregnancy complications, maternity leave, or disease/death in the family) was omitted from that month as the absolute number of home visits would not reflect performance.
** p<0.01, * p<0.05

Appendix S1

Description of the CHW-led health system strengthening intervention

In Yirimadio, a CHW-led health systems strengthening intervention was jointly launched in 2008 by a nongovernmental organisation and the Malian Ministry of Health and Public Hygiene. At that time, 20 CHWs were trained and deployed in non-overlapping intervention zones covering all of Yirimadio to provide health services without user fees to patients in their homes. In 2013, to keep up with population growth and maintain CHW-to-population ratio of approximately 1:1000, a second wave of CHWs were recruited and trained, bringing the total to 75. Successful candidates were members of the community, could read and write in the local language (Bamanankan), and priority was given to women. At this time, individual monthly supervision was introduced, with the four highest-scoring CHWs on written exams promoted to positions of dedicated CHW supervisor and trained for the exclusive purpose of supervising 15 to 20 CHWs under his/her supervision.

By October 2015, the population of Yirimadio had nearly doubled and a third wave of recruitment brought the total to 150 CHWs with four additional supervisors. The four additional supervisors were all external recruits with an education level of at least two years after Baccalaureate, experience in supervision and working in a team, and basic informatics skills. At each wave of recruitment, both new and old supervisors and CHWs were trained by the nongovernmental organisation on service provision and data collection. All eight supervisors and 150 CHWs participated in the current study: 148 CHWs were study participants providing written informed consent, while two CHWs pretested the performance dashboard during the design of the tool.

During the study period (October 2015 to June 2016), all CHWs, regardless of treatment arm, conducted at least two hours per day of door-to-door home visits to proactively identify patients who need care. For all patients identified through this search, CHWs provided doorstep counselling, evaluation, diagnostics, treatment, referral to appropriate health facilities, and follow-up and removed user fees for all services provided. CHWs administered doorstep community case management for simple cases of malaria, diarrhoea, pneumonia and malnutrition to children under five (2-59 months for malaria, diarrhoea and pneumonia; 6-59 months for acute moderate malnutrition), rapid referral to the government primary health centre (PHC) when higher care was needed, and at-home follow-up at 24, 48, and 73 hours, with an additional five days follow-up for diarrhoeal cases. For women of reproductive age, CHWs provided doorstep pregnancy testing, facilitated early access to prenatal care and facility-based delivery if pregnant, same-day access to contraception if not pregnant, and follow-up during and after pregnancy. CHWs also conducted newborn assessment in the home during the days and weeks following childbirth, screened for danger signs and accompanied mother and child to the PHC if danger signs were detected. For all other sick patients and for cases beyond the CHW's scope of work, the CHW provided referral or accompaniment to the PHC. In order to ensure quality referral care, the PHC also received improved infrastructure, equipment, and capacity building training for staff. Finally, the CHW used behaviour change communication to promote healthy living among families and prevent disease.
